# Weight Changes in Women Receiving Chemotherapy for Non-Metastatic Breast Cancer in Saudi Arabia

**DOI:** 10.7759/cureus.12961

**Published:** 2021-01-28

**Authors:** Marwan Al-Hajeili, Nora Trabulsi, Manar A Makin, Noor Shibriq, Reem Alshelali, Lubna Alghoraibi, Raneem Alhaidari, Lujain Alhazzani, Amani S Alzahrani

**Affiliations:** 1 Oncology, King Abdulaziz University Hospital, Jeddah, SAU; 2 General Surgery, King Abdulaziz University, Jeddah, SAU; 3 Faculty of Medicine, King Abdulaziz University, Jeddah, SAU; 4 Dermatology, King Abdulaziz University, Jeddah, SAU; 5 Medical Physics, King Abdulaziz University, Jeddah, SAU

**Keywords:** saudi arabia, chemotherapy, breast cancer, weight gain, weight loss

## Abstract

Background

Women with breast cancer (BC) commonly experience weight gain during chemotherapy, although there is conflicting evidence regarding the contributing factors. This study aimed to evaluate body weight changes among women undergoing chemotherapy for non-metastatic BC during the first year after diagnosis, and to determine whether baseline body weight and/or hemoglobin concentration values were associated with weight changes during chemotherapy.

Methods

This retrospective study evaluated patients who were treated at the King Abdulaziz University Hospital (Saudi Arabia) during 2010-2019. A total of 228 women were included based on the following criteria: new diagnosed BC, age of 18-80 years, non-metastatic disease, and initial chemotherapy treatment for BC. The patients’ baseline characteristics, including body weight during the first chemotherapy cycle, were collected from their electronic medical records. Each patient’s weight was then followed at each hospital visit until the last chemotherapy cycle. In addition, data were collected regarding tumor status, menopausal status, chemotherapy regimen, hemoglobin concentration, recurrence status, and death.

Results

The mean patient age was 53.37±10.9 years and 55.7% of the patients were pre-menopausal. The vast majority of patients underwent surgery (96.9%) and most patients received adjuvant chemotherapy (63.6%) or adjuvant radiotherapy (68.9%). The mean number of chemotherapy cycles was 6.29±1.74 (taxane-based: 1.67±1.36 cycles, anthracycline-based: 2.61±1.81 cycles). At the end of chemotherapy, the body weight changes were classified as increased (41.7% of patients, mean increase: 3.39 kg), decreased (35.5% of patients, mean decrease: -4.12 kg), or stable (22.8%). Factors that predicted weight gain after chemotherapy included younger age at diagnosis (p<0.029), pre-menopausal status (p<0.003), and a high number of taxane-based chemotherapy cycles (p<0.029).

Conclusions

Chemotherapy for BC did not lead to significant changes in body weight among women in Saudi Arabia. Weight gain in this setting was significantly associated with younger age, pre-menopausal status, and a high number of taxane-based chemotherapy cycles.

## Introduction

Breast cancer (BC) is the most common invasive cancer and affects 2.1 million women worldwide [1]. In Saudi Arabia, BC is considered the most common malignancy among women and accounts for 17.3% of all cancer types and >30% of newly diagnosed cancer cases [2]. Management of BC requires a multimodal strategy that involves surgery, radiotherapy, and systemic therapy. A large proportion of women with BC in Saudi Arabia will receive chemotherapy as part of their treatment strategy, based on their generally advanced disease and young age at diagnosis [3].

Chemotherapy for BC is associated with various side effects, including nausea, vomiting, diarrhea, bone marrow suppression, muscle weakness, fatigue, dry mouth, anorexia, emotional/psychological disturbances, and rapid onset of menopause [4,5]. Chemotherapy is also associated with metabolic changes, including weight changes [6]. There is conflicting evidence regarding the changes in body weight during and after chemotherapy for BC. Some studies have identified significant weight gain during and after chemotherapy [7], while other reports have only identified weight gain during chemotherapy [8]. Multiple factors could contribute to this phenomenon, including stress, overeating to alleviate nausea, decreased physical activity because of fatigue, concomitant corticosteroid use, and early onset of menopause [9]. Hemoglobin concentrations may also influence weight changes because of their effects on physical functioning, mood, and quality of life [10]. However, patients are thought to have the highest risk of weight gain during the pre-menopausal phase [11]. In addition, the patient’s baseline weight can influence chemotherapy selection [12] and the chemotherapy dose may be decreased for overweight or obese patients (vs. patients of average weight) [12,13].

A meta-analysis has revealed that newer chemotherapy regimens, such as taxane- and anthracycline-based regimens, were associated with less weight gain, relative to older regimens that included cyclophosphamide, methotrexate, and fluorouracil [14]. The same meta-analysis also identified a weight gain of 2.0-7.5 kg during chemotherapy in the first year following a BC diagnosis [14]. Moreover, BC patients who gain weight during their treatment are unlikely to return to their pre-diagnosis weight [15]. Despite this information, there are limited data regarding changes in body weight among Saudi women who receive chemotherapy for BC. As risks of future adverse events are related to weight gain and obesity. It would be useful to identify factors that predicted weight gain in this patient population and to develop appropriate education strategies. Therefore, this study aimed to evaluate weight changes among women who were receiving chemotherapy for non-metastatic BC during the first year after their diagnosis, and to determine whether baseline body weight and/or hemoglobin concentration values were associated with weight changes during chemotherapy.

## Materials and methods

This retrospective descriptive study evaluated 228 women who were treated for non-metastatic BC at the King Abdulaziz University Hospital (Jeddah, Saudi Arabia) during 2010-2019. The inclusion criteria were women who were 18-80 years old, a new diagnosis of non-metastatic BC, and initial chemotherapy treatment for BC. Patients were excluded if they had metastatic disease at their presentation, received chemotherapy for cancer at another hospital, or had no weight records. The retrospective protocol was approved by the research ethics committee of King Abdulaziz University Hospital, which waived the requirement for informed consent. The principal investigators and co-investigators stored anonymized patient data on password-secured personal computers.

The data were collected from hospital records. Patients’ weight, height, and body mass index were collected and documented by trained nurses at each chemotherapy session. The baseline values were defined as the values from the first chemotherapy cycle, and follow-up values were collected at the start of each subsequent chemotherapy cycle. All patients completed their chemotherapy cycles over a period of less than one year. Stable weight was defined as a gain or loss of less than 1kg, and changes in weight were defined as more than 1kg. Patients visited an outpatient clinic for hemoglobin testing at one to three days before the start of each chemotherapy cycle, and hemoglobin concentrations were extracted from the patients’ medical records. In addition, data were extracted regarding tumor status, menopausal status, chemotherapy regimen(s), recurrence status, and death.

All statistical analyses were performed using SPSS software (version 24; IBM Corp., Armonk, NY). Categorical data were expressed as number (percentage) and evaluated using the χ2 test. Continuous data were expressed as mean ± standard deviation and evaluated using the t-test or Wilcoxon test, as appropriate. Results were considered statistically significant at two-tailed p-values of <0.05.

## Results

Table 1 shows the patients’ clinicopathological characteristics. The mean patient age was 53.37±10.9 years, 48.7% of patients were of Saudi nationality, and 55.7% of patients were pre-menopausal. Common disease characteristics were T2 classification (53.9%), N1 classification (40.4%), estrogen receptor-positive (66.2%), progesterone receptor-positive (62.7%), human epidermal growth factor receptor 2-positive (36.8%), and triple-negative status (18.9%).

**Table 1:** The clinicopathological characteristics of the 228 patients. Data are presented as mean ± standard deviation or number (%).

	Results
Age, years	53.37±10.9
Nationality	
Non-Saudi	117 (51.3)
Saudi	111 (48.7)
Menopausal status	
Post-menopausal	101 (44.3)
Pre-menopausal	127 (55.7)
T classification	
1	60 (26.3)
2	123 (53.95)
3	19 (8.3)
4	13 (5.7)
Unknown	13 (5.7)
N classification	
0	84 (36.8)
1	92 (40.4)
2	32 (14)
3	11 (4.8)
Unknown	9 (3.9)
M classification	
0	228 (100)
Estrogen receptor	
Negative	76 (33.3)
Positive	151 (66.2)
Unknown	1 (0.4)
Progesterone receptor	
Negative	84 (36.8)
Positive	143 (62.7)
Unknown	1 (0.4)
Human epidermal growth factor receptor 2	
Equivocal	3 (1.3)
Negative	140 (61.4)
Positive	84 (36.8)
Unknown	1 (0.4)
Triple-negative	
Yes	43 (18.9)
No	185 (81)

Table 2 shows the treatment-related variables. Almost all patients underwent surgery (96.9%) and most patients received adjuvant chemotherapy (63.6%) or adjuvant radiotherapy (68.9%), with the remaining patients receiving neoadjuvant chemotherapy. The mean number of chemotherapy cycles was 6.29±1.74 (taxane-based: 1.67±1.36 cycles, anthracycline-based: 2.61±1.81 cycles), and most patients received anthracycline- or taxane-based chemotherapy (60.1%). Paused or stopped chemotherapy was observed for 11.4% of patients. The mean duration of chemotherapy (first to last cycle) was 3.69±1.99 months. During a median follow-up period of two years, 10 patients (4.4%) experienced local and/or distant recurrence and four patients (1.8%) died.

**Table 2:** Relationships between weight changes and the patients’ clinical and treatment characteristics. Data are presented as number (%) or mean ± standard deviation.

	Weight change			Wilcoxon test	p-value
	Increased	Decreased	Stable		
Nationality					
Non-Saudi	51 (43.6)	37 (31.6)	29 (24.8)	1.65	0.437
Saudi	44 (39.6)	44 (39.6)	23 (20.7)		
Menopausal status					
Post-menopausal	31 (30.7)	38 (37.6)	32 (31.7)	11.72	0.003
Pre-menopausal	64 (50.4)	43 (33.9)	20 (15.7)		
Type of treatment					
Adjuvant	60 (41.4)	48 (33.1)	37 (25.5)	1.94	0.377
Neoadjuvant	35 (42.2)	33 (39.8)	15 (18.1)		
Adjuvant radiotherapy					
No	28 (39.4)	28 (39.4)	15 (21.1)	0.69	0.707
Yes	67 (42.7)	53 (33.8)	37 (23.6)		
Chemotherapy stopped or paused					
No	85 (42.1)	72 (35.6)	45 (22.3)	0.29	0.862
Yes	10 (38.5)	9 (34.6)	7 (26.9)		
Recurrence after surgery					
No	90 (41.3)	79 (36.2)	49 (22.5)	1.12	0.571
Yes	5 (50)	2 (20)	3 (30)		
Death					
No	94 (42)	79 (35.3)	51 (22.8)	0.52	0.771
Yes	1 (25)	2 (50)	1 (25)		
Age, years	50.71±10.74	53.69±10.11	57.69±11.13	2	0.029
Taxane-based cycle	3.89±1.11	3.84±1.07	3±1.87	2	0.029
Anthracycline-based cycles	2.7±1.78	2.64±1.83	1.42±1.88	2	0.915
Total chemotherapy cycles	6.5±1.63	6.56±1.6	5.48±1.91	2	0.504
Duration of chemotherapy	3.21 ± 1.6	3.22 ± 1.	5.3 ± 2.3	2	0.83

**Figure 1:**
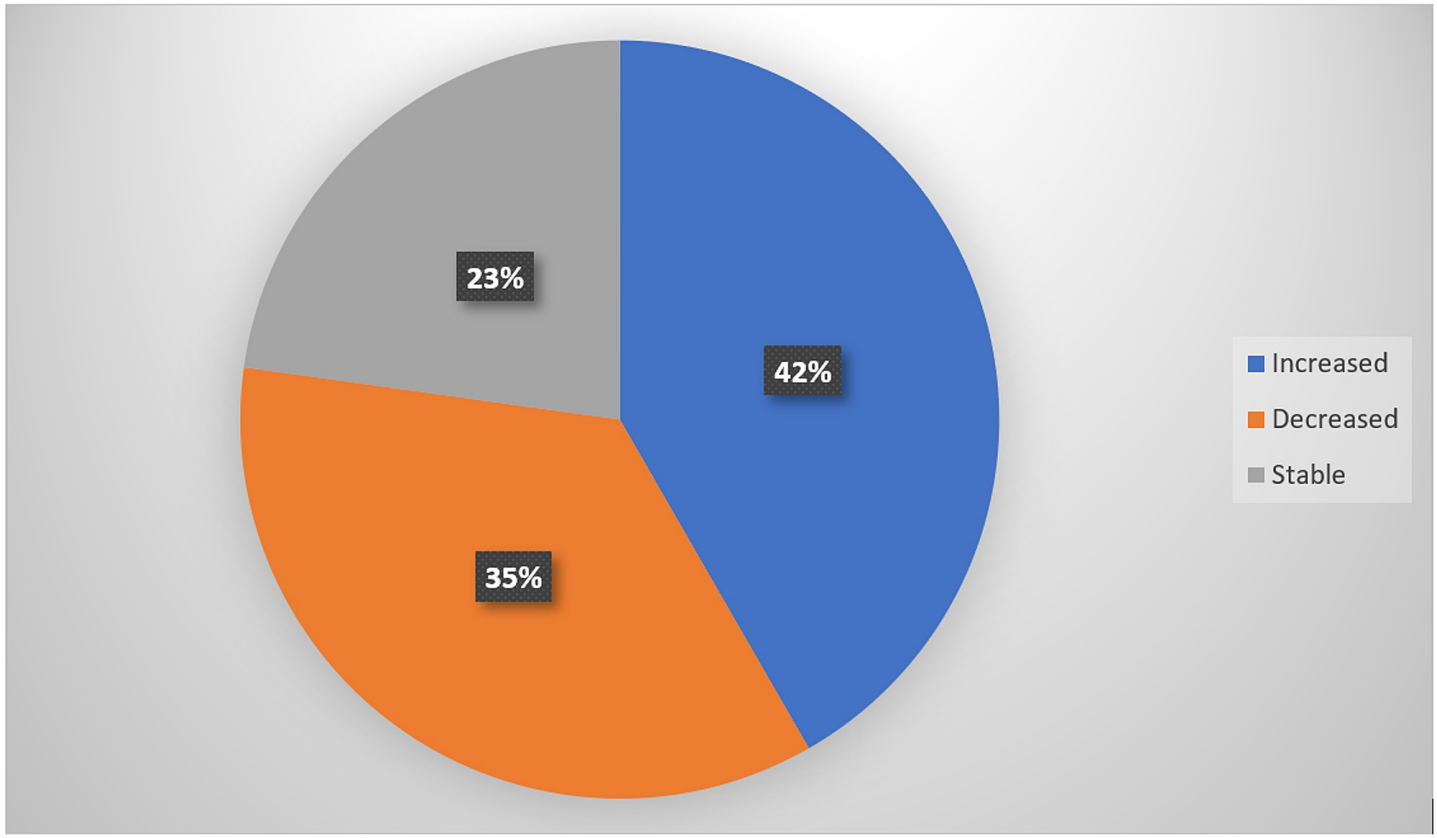
Distributions of weight changes during chemotherapy.

Relative to the baseline weight, weight changes were classified as stable weight (22.8% of patients), increased (41.7% of patients), or decreased (35.5%) (Figure 1). The mean weight gain was 3.39±2.89 kg (range: 0.5-17 kg) and the mean weight loss was 4.12±3.97 kg (range: 0.5-17.4kg). Weight gain was significantly associated with younger age at diagnosis (p<0.029), pre-menopausal status (p<0.003), and high number of taxane-based chemotherapy cycles (p<0.029). Weight changes were not significantly associated with tumor status, hemoglobin concentration, hormonal receptor status, or surgical status (Table 3). A non-significant relationship was observed between the patients’ baseline weight and post-chemotherapy weight (p>0.05) (Table 4).

**Table 3:** Treatment characteristics and survival outcomes. Data are presented as number (%) or mean ± standard deviation.

	Results
Type of treatment	
Adjuvant	145 (63.6)
Neoadjuvant	83 (36.4)
Adjuvant radiotherapy	
No	71 (31.1)
Yes	157 (68.9)
Surgery	
No	7 (3.1)
Yes	221 (96.9)
Chemotherapy paused or stopped	
No	202 (88.6)
Yes	26 (11.4)
Recurrence after surgery	
No	218 (95.6)
Yes	10 (4.4)
Death	
No	224 (98.2)
Yes	4 (1.8)
Weight change during chemotherapy	
Increased	95 (41.7)
Decreased	81 (35.5)
Stable	52 (22.8)
Chemotherapy parameters	
Anthracycline-base	20 (8.8)
Taxane-based	71 (31.1)
Anthracycline plus taxane	137 (60.1)
Taxane-based cycles	1.67±1.36
Anthracycline-based cycles	2.61±1.81
Total chemotherapy cycles	6.29±1.74
Duration of chemotherapy, months	3.69±1.99

 

**Table 4:** Relationships between baseline and post-chemotherapy values for weight and body mass index. Data are presented as mean ± standard deviation. BMI: body mass index.

	Weight (kg)	Wilcoxon test	p-value
Cycle 1, reference	73.50±17.17		
Cycle 2	73.50±17.17	0.52	0.599
Cycle 3	73.30±17.04	1	0.314
Cycle 4	73.28±17.35	0.26	0.79
Cycle 5	73.10±16.54	0.23	0.814
Cycle 6	73.53±16.79	1.52	0.126
Cycle 7	72.29±15.95	1.28	0.2
Cycle 8	71.64±15.92	1.13	0.257
	BMI (kg/m^2^)		
Cycle 1, reference	30.84±6.6		
Cycle 2	30.78±6.47	0.69	0.488
Cycle 3	30.69±6.42	1.27	0.22
Cycle 4	30.67±6.5	0.29	0.769
Cycle 5	30.59±6.16	0.32	0.749
Cycle 6	30.59±6.79	1.21	0.215
Cycle 7	30.27±5.92	1.13	0.257
Cycle 8	29.59±6.46	0.95	0.341

## Discussion

This study evaluated weight changes during chemotherapy among women with non-metastatic BC in the first year after their diagnosis, which remains a poorly understood issue in Saudi Arabia. This retrospective study at a center in Saudi Arabia revealed no significant weight gain or loss during chemotherapy with 77.2% of the patients experienced a >1-kg change in their weight during chemotherapy. Which was classified as an increase for 41.7% of patients or a decrease for 35.5% of patients. These results are similar to those of a previous retrospective study by Wang et al., which identified weight gain in 34.6% of patients and weight loss in 32.1% of patients [16]. Another prospective study by Freedman et al. identified weight gain in 25% of patients and weight loss in 20% of patients [17]. These inter-study differences might be related to differences in sample size (i.e. 26 patients vs. our 228 patients) and the period over which weight change was calculated, as we calculated the weight change at the end of chemotherapy and the prospective study continued follow-up for 6 months after chemotherapy. A meta-analysis has indicated that the mean weight change varied from −0.8kg to +7.7kg [14], while our results revealed weight loss of 0.5-17.4kg and weight gain of 0.5-17kg. Our results might reflect a general lack of health awareness among Saudi patients, who may have an unhealthy lifestyle that leads to weight gain [18]. However, a Korean study has revealed that most patients did not experience substantial weight changes after chemotherapy [19], which may suggest that weight changes in this setting are related to nation-specific cultural values and special dietary habits. Hospital-based education may play a role in helping manage weight changes in this setting [19].

Several factors may contribute to weight gain following adjuvant chemotherapy, such as age, baseline weight, menopausal status, chemotherapy regimen, hormonal receptor status, disease stage, and a number of chemotherapy cycles. In addition, weight gain may be influenced by lifestyle, education level, and economic status [20]. The present study revealed that younger women (mean age: 50.71±10.74 years) had a higher likelihood of weight gain, which is consistent with a previous report by Wang et al. the multivariable logistic regression indicated that age of equal or less than 40 years is independently associated with an increased likelihood of weight gain [16]. Another two studies had demonstrated similar findings too [19,21].

The association between menopausal status and weight change has remained controversial for several years [22]. Goodwin et al. evaluated weight gain during the first year of treatment for 535 women with newly diagnosed BC, and reported that post-menopausal status independently predicted weight gain [9]. However, another population-based cohort study evaluated 4,561 women with BC at 18, 36, and 60 months after their diagnosis, and multinomial logistic regression analysis revealed that pre-menopausal women gained more weight than post-menopausal women [19]. This finding also agrees with our results, as we observed that pre-menopausal status was associated with more weight gain during chemotherapy (vs. post-menopausal status). Goodwin et al. noted that the greatest weight gain (mean: +2.62kg) was observed among patients who entered menopause during chemotherapy, which may indicate that estrogen deficiency contributes to subsequent weight gain [9]. Campbell et al. have also reported that pre-menopausal women might be prone to develop amenorrhea or premature menopause during cancer treatment, which might increase their likelihood of gaining weight, relative to post-menopausal women [22].

A meta-analysis of 2,620 women from 25 reports (published between 1985 and 2015) revealed that reports published after 2000 identified less weight gain (1.4-5kg), relative to older reports (2.5-6.2kg). This might be related to changes in chemotherapies that are used for BC, as cyclophosphamide, methotrexate, and fluorouracil were common before the 1990s, although taxane-based regimens have become more common since that time [14]. The present study revealed that the patients received taxane-based chemotherapy (31.1%), anthracycline-based chemotherapy (8.8%), or taxane- plus anthracycline-based chemotherapy (60.1%). Furthermore, a higher number of taxane-based chemotherapy cycles (mean: 3.89±1.11 cycles) was significantly associated with weight gain. Another study evaluated 119 patients who completed neoadjuvant chemotherapy with at least two years of follow-up (median: 26.3 months) and revealed that, among 97 patients who received anthracycline followed by taxane, the mean weight changes were -0.8 kg after anthracycline and +3.2kg after taxane [23], which are similar to our results. Another multicenter retrospective study (2001-2010) revealed that treatment using anthracycline plus taxane was associated with more weight gain (+0.9kg) relative to anthracycline alone, which the authors attributed to the longer duration of the combination treatment [24].

The present study has several limitations. First, the single-center design may preclude generalization of the findings to all women with BC in Saudi Arabia. Second, the retrospective design made it impossible to collect data regarding factors that may affect weight change, such as dietary habits, physical activity, psychological status, and education. Third, random measurement errors might be an influence, given the different timing and techniques for body weight measurements. Nevertheless, this study also has several strengths. First, patients were treated at a center that specializes in treating BC, which is located in Jeddah City (a major city in Saudi Arabia). Second, this is one of the first studies to examine weight changes among patients with BC in Saudi Arabia.

## Conclusions

This study at a Saudi Arabian hospital evaluated weight changes during chemotherapy in women with non-metastatic breast cancer within the first year after their diagnosis. The results revealed no significant weight gain or loss during chemotherapy. Among women who had weight gain, factors that were associated with weight gain were pre-menopausal status, younger age, and a high number of taxane-based chemotherapy cycles. Large prospective studies with more chemotherapy regimens, prolonged follow-up periods and lifestyle measures such as dietary habits and physical activity are needed to clarify the prognostic relevance of weight gain in this setting, which might help guide more aggressive strategies for controlling weight during chemotherapy.
